# Compositional analysis of natural pomegranate peel powder dried by different methods and nutritional and sensory evaluation of cookies fortified with pomegranate peel powder

**DOI:** 10.3389/fnut.2023.1118156

**Published:** 2023-03-14

**Authors:** Ali Muhammad, Kenan Sinan Dayisoylu, Jinjin Pei, Muhammad Rafiullah Khan, Muhammad Salman, Rafiq Ahmad, Hakim Ullah, Gul Rah Noor

**Affiliations:** ^1^Shaanxi Province Key Laboratory of Bioresources, 2011 Qinling-Bashan Mountains Bioresources Comprehensive Development C. I. C., Qinba State Key Laboratory of Biological Resources and Ecological Environment, College of Bioscience and Bioengineering, Shaanxi University of Technology, Hanzhong, Shaanxi, China; ^2^Department of Food Science and Technology, The University of Agriculture, Peshawar, Pakistan; ^3^Department of Food Engineering, Kahramanmaraş Sütçü İmam University, Kahramanmaraş, Türkiye; ^4^Department of Food Engineering, Pak-Austria Fachhochschule: Institute of Applied Sciences and Technology, Haripur, Pakistan; ^5^Department of Microbiology, The University of Haripur, Haripur, Pakistan; ^6^Department of Plant Breeding and Genetics, The University of Agriculture, Peshawar, Pakistan

**Keywords:** pomegranate peel powder, cookies, drying methods, mineral profile, proximate composition, wheat flour, fortification

## Abstract

**Introduction:**

Fortification of cereal products with natural plant extract is an interesting approach to fulfill the dietary requirement of the people.

**Materials and methods:**

Peels of pomegranate (rich source of natural compounds) were cut into small pieces and dried in three different methods such as solar drying (SOD), oven drying (OD), and sun drying (SUD). The fine powder was prepared and proximate compositions (protein, ash, moisture, fats, fiber, and carbohydrates), minerals (zinc, iron, calcium, and potassium), total phenolic content (TPC), total flavonoid content (TFC), and antioxidant activity (DPPH) of the pomegranate peel powder (PP) were evaluated. Fine wheat flour (FWF) was fortified with different concentrations (3, 6, 8, 10, and 12 g) of PP powder, cookies were prepared and all the above analysis along with physical parameters (weight, width, thickness, spread ration) and sensory analysis were conducted. Cookies without PP powder were served as control.

**Results and discussion:**

Results showed that a SOD was the best for drying PP powder in terms of compositional analysis. Addition of PP powder significantly (*P* < 0.05) enhanced the nutritional value, minerals profile and physical attributes of the fortified cookies. Sensory analysis of fortified cookies indicated that the cookies were acceptable to the sensory panel. Therefore, in conclusion, PP powder dried by SOD method could be used commercially in baking industries to provide nutritional enriched cookies to fulfill the dietary requirements of the people.

## Introduction

Among all the horticultural commodities fruits and vegetables are highly consumed and produce waste materials in the form of peels and seeds. In juice industry peels and seeds of pomegranate fruits are remained as a byproducts and considered as waste which leads to environmental pollutions (1). These wastes are good sources of potential compounds for the well-being of nourishment and malnutrition. These bioactive compounds can be utilized as source of medicines, pharmaceutical purpose, source of fibers and a step toward economic growth (2).

Pomegranate fruit contains a significant percentage of phenolic contents. The peel of fruit (representing 50% of the fruit by weight) contain high amount of bioactive substance such as tannins content, flavonoids and phenolic contents with antimicrobial activity. These constituents possess radical scavenging properties and prevent lipid oxidation in fatty foods (3, 4). Pomegranate peel contains biologically active substances such as Anthocyanins, phytochemicals, organic acids, quercetin, catechin, gallic acid, caffeic acid, antioxidants, minerals and vitamins (5, 6) and abundant potential benefits. These compounds are very important for health and can protect against some deteriorating diseases, such as (CVD) diseases, infections, infarcts, diabetes, and cancers (4).

Pomegranate production exceeds 2 million tons per year around the world (7). The climatic condition of turkey is extremely suitable for the cultivation of pomegranate cultivars. The registered cultivars that are grown around the world exceed 30 cultivars while there are serval local cultivars that are famous and grown by local orc handlers (8). Pakistan ranked 10th in the production of pomegranates with an annual production of approximately 57,800 tons on an area of 14,900 hectares. However, the production rate is quite low (3.88 tons ha^–1^). The cultivated cultivar of pomegranate in Pakistan are Sandhora, Sava (white arils), Kalahari (Pinkish white arils), Be-Danna (soft seeded red and white arils), Kandahari, and Tarnab-Gulabi. As it is an ancient fruit crop having a relation to the different warm temperate climate and is commercially produced in regions of several climatic zones, there is limited research on ecological, structural changes and the germplasm characterization (9).

Cereal products, particularly bakery products, are consumed in breakfast almost all over the world. Some bakery products, particularly cookies, are generally used as major snack products and are prepared from starches, a refined portion of wheat flour. However, during the milling process of wheat flour, removal of the bran, and another operation process, nutrients and or mineral deficiency occurs, which needs to be fortified to fulfill the dietary requirements of the people (10, 11).

Fortification of cereals by natural means particularly plant-based compounds is one of the best approaches. Peel of the pomegranate fruit contains a high number of bioactive substances and plays a significant role in the curing of various diseases. Pomegranate peel contains a natural source of dietary fiber ranging from 33 to 62 percent. The highest concentration of fibers in peel is lignin, followed by cellulose (16–22%) (12). Pomegranate peels are widely used as food stabilizers in production of alginate microsphere with high antioxidant activity than commercially available in the market (13).

Among the bakery products, cookies represent the largest category of snack items (14). Snacks are one of the famous food due to its affordable price, nutritive value, convenience and shelf stability for both young and elderly peoples (15). Cookies are acceptable baked products and consumed in many countries by all profiles of consumers (16). Cookies are generally prepared from refined flour, hydrogenated fat, sugar, emulsifier and some minor food additives (17). Its nutritional values can be improved by enrichment and supplementation process by addition of non- wheat flour (18). The fortified cookies are commonly prepared from oyster mashroom, water chest nut, cassava, composite flour, wheat germ and other source of nutrients such as whey protein concentrate to increase its nutritive value and demand (16–19). Pomegranate peel can enhance the flavonoid and anthocyanin contents of the cookies and could improve the nourishing quality, sensorial, rheological, and antioxidant activities (20).

Therefore, in this study fine wheat flour was fortified with different concentrations of PP powder and cookies were prepared to improve their nutritional and therapeutic value of cookies. The cookies fortified with PP powder could increase the fibers, and minerals and consequently will have a positive impact in reduction of osteoporosis, anemia, and other diseases. This fortification approach is one of the cost-effective and easy way to eradicate these diseases.

## Materials and methods

### Reagents

Folin–Ciocalteu’s phenol reagent, phosphoric acid and hydrogen peroxide, 2,2-diphenyl-1-picrylhydrazyl (DPPH), methanol HPLC grade, were obtained from Merck Darmstadt, Germany. Gallic acid and Quercetin, (98% HPLC grade), were purchased from Sigma Chemical Co., (St. Louis, MO, USA).

### Preparation of PP powder

Pomegranate cv. Kandahari, were obtained from the local market of Peshawar city, Khyber Pakhtunkhwa Province and after preparatory operations, the peels were cut into small pieces and dried by three different methods such as solar drying (SOD) at 50°C with air flow rate of 4.72 Kgm^–1^ for 12 h, oven (WTB binder, Tuttlingen, Germany) drying (OD) at 65°C for 24 h and sun drying (SUD) dried for 72 h. Then powder was prepared by a grinding machine (ZM 200, Retsch, Haan, Germany) and sieved through a 50-mesh type (*297 micron*) to obtain a uniform fine PP powder.

To select the best drying method and high nutritional peel powder for cookie fortification, all the peels were evaluated for their proximate analysis, minerals profile, total phenolic content, total flavonoid contents and antioxidant activities as follows.

### Determination of proximate composition of PP powder and FWF

The moisture content, ash, crude protein, crude fat, crude fiber, and nitrogen free extract (NFE) of PP powder, FWF and prepared cookies, were determined according to the method of AACC (21) with the following respective formulas.


(1) $$Moisture\left(\%\right)=\frac{I\,n\,i\,t\,i\,a\,l\,s\,a\,m\,p\,l\,e\,w\,e\,i\,g\,h\,t-F\,i\,n\,a\,l\,s\,a\,m\,p\,l\,e\,w\,e\,i\,g\,h\,t}{I\,n\,i\,t\,i\,a\,l\,s\,a\,m\,p\,l\,e\,w\,e\,i\,g\,h\,t}\times 100$$

(2) $$Ash\left(\%\right)=\frac{I\,n\,i\,t\,i\,a\,l\,s\,a\,m\,p\,l\,e\,w\,e\,i\,g\,h\,t-F\,i\,n\,a\,l\,s\,a\,m\,p\,l\,e\,w\,e\,i\,g\,h\,t}{I\,n\,t\,i\,t\,i\,a\,l\,s\,a\,m\,p\,l\,e\,w\,e\,i\,g\,h\,t}\times 100$$

(3) $$Crudeprotein\left(\%\right)=\frac{\left(T\,R-B\right)\times N\,o\,r\,m\,a\,l\,i\,t\,y\,a\,n\,d\,a\,c\,i\,d\,u\,s\,e\,d\times 0.014\times 20\times 100}{W\,e\,i\,g\,h\,t\,o\,f\,s\,a\,m\,p\,l\,e}\times 100$$

Where TR is titration reading, B = blank, 0.014 is milliequivalent weight of Nitrogen, 20 = dilution, conversion factor of protein = 6.25, and 100 = volume.


(4) $$Crudefat\left(\%\right)=\frac{F\,i\,n\,a\,l\,s\,a\,m\,p\,l\,e\,w\,e\,i\,g\,h\,t\,\left(W\,2\right)-I\,n\,i\,t\,i\,a\,l\,s\,a\,m\,p\,l\,e\,w\,e\,i\,g\,h\,t\,\left(W\,1\right)}{I\,n\,i\,t\,i\,a\,l\,s\,a\,m\,p\,l\,e\,w\,e\,i\,g\,h\,t}\times 100$$

Where W1 and W2 are the initial weight of sample before fat extraction and final weight of sample after fat extraction.


(5) $$Crudefiber\left(\%\right)=\frac{W\,2-W\,3}{W\,1}\times 100$$

Where W1 = Sample weight. W2 = crucible weight with fiber and ash after drying in oven. W3 = crucible weight with ashes after muffle furnace. Nitrogen free extract which is called carbohydrate was find out by the differential method:


(6) NFE(%)=

100-(%moisture+%ash+%fats+%protein+%fiber)

### Determination of mineral analysis of PP powder and FWF

Minerals analysis (calcium, potassium, iron, and zinc) of PP powder, FWF and cookies were determined according to the method of Khan et al. (22). Briefly, wet digestion method was used by mixing 0.2 g of sample in 10 mL concentrated nitric acid overnight, then 4 mL of per chloric acid was added and samples were heated for digestion. Digested samples were cooled at ambient temperature, filtered and analysis were carried out using UV spectrophotometer (Model U-1800 Hitachi, Japan) and atomic absorption (Model AA-6300, Shimadzu, Kyoto, Japan).

### Determination of total phenolic content in PP powder

The TPC in PP powder was determined by UV spectrophotometer (Model U-1800 Hitachi, Japan) at 765 nm, using Folin-Ciocalteu reagent according to the method of Zainol et al. (23). Results for TPC were expressed as a gram of gallic acid per kg (mg GAE g^–1^) of fresh weight ± SD. The experiment was done in triplicate.

### Determination of total flavonoid content in PP powder

The TFC in PP powder was measured by the method of Adom et al. (24) and Muscolo et al. (25) using spectrophotometric (Model U-1800 Hitachi, Japan) at 510 nm. Results for TFC were expressed as a gram of quercetin per gram (mg quercetin g^–1^) of fresh weight ± SD. The experiment was done in triplicate.

### Determination of DPPH free radical scavenging activity of PP powder

2,2-diphenyl-1-picrylhydrazyl (DPPH) radical is based on the measurement of the scavenging ability of antioxidants substances toward the stable radical and measured by the method of Pownall et al. (26) using the following formula.


(7) D⁢P⁢P⁢H=[1-(A⁢B⁢A⁢s⁢a⁢m⁢p⁢l⁢e/A⁢B⁢S⁢c⁢o⁢n⁢t⁢r⁢o⁢l)]×100

### Preparation of PP powder fortified cookies

**FIGURE 1:**
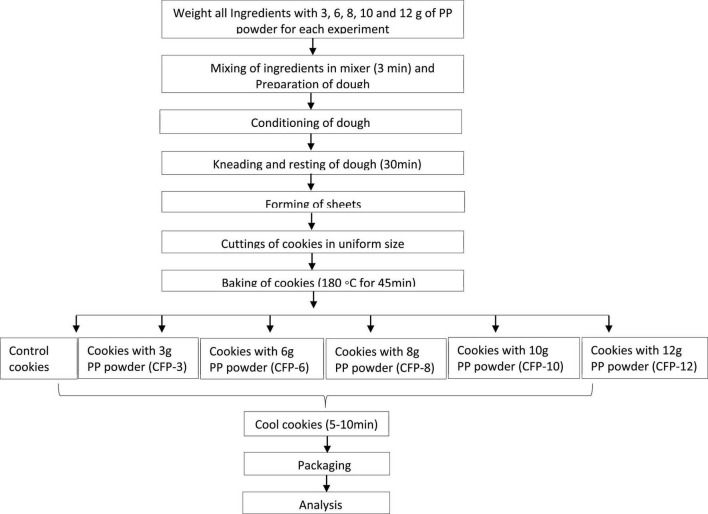
Flow diagram of cookies preparation and respective analysis.

Cookies were prepared by following the method of Zaker et al. (27) with slight modification. Briefly, a mixture of 160 g of FWF, 62.5 g of sugar, 62.5 g of butter, 1.25 g of baking powder and one forth part of egg were mixed homogeneously and served as control treatment (without PP powder). While for the fortification purposes, different concentrations of PP powder were used, i.e., 157 g of FWF + 3 g of PP powder, 154 g of FWF + 6 g of PP powder, 152 g of FWF + 8 g of PP powder, 150 g of FWF + 10 g of PP powder and 148 g of FWF + 12 g of PP powder. Other ingredients like sugar, butter, baking powder and egg were used kept constant as that in control treatment. All the ingredients were mixed thoroughly to obtain homogeneous paste or dough and were cut into round shape cookies of the uniform size. These were then kept in oven at 175°C for 15 min. Then cookies were packed in airtight container for further analysis. Each treatment was conducted in triplicate. The flow diagram of cookies preparation is presented in Figure 1.

### Quality evaluation of PP powder fortified cookies

#### Determination of proximate composition and mineral profile of PP powder fortified cookies

Proximate composition such as moisture, ash, crude fat, crude protein, crude fiber, NFE, and minerals such as calcium, potassium, iron, and zinc were analyzed as discussed above.

#### Determination of physical attributes of PP powder fortified cookies

Physical attributes such as weight was measured by the method of Ayo et al. (28). The width and thickness (mm) of cookies were measured by a vernier caliper according to the protocol of AACC (21). Spread ratio was measured by dividing the width by thickness.

#### Sensory evaluation of PP powder fortified cookies

Sensory analysis such as color, texture, taste and overall acceptability of cookies were conducted by 25 semi trained judges (13 males and 12 females) age limit from 25 to 30 years with no addicted drug history, selected from department of Food Science and Technology, as described by Ho and Latif (29) and Bhalerao et al. (30) with a little modification, using 9-points hedonic scale (1 = extremely dislike, 2 = very much dislike, 3 = moderately dislike, 4 = dislike-slightly, 5 = neither-like nor-dislike, 6 = like-slightly, 7 = like-moderately, 8 = like-very much, 9 = like-extremely) proposed by Larmond (31).

### Statistical analysis

Data were statistically analyzed by Completely Randomized Design (CRD), with 2-factorials, using Statistix 8.1 software, Tallahassee, Florida, USA. Means were separated by least significant difference (LSD) test at *P* ≤ 0.05 to find the significance level among the treatments. The results obtained from the present study are represented as the mean values of three replicates ± standard deviation (SD).

## Results and discussion

### Proximate composition of PP powder

Proximate compositions of solar, oven and sun-dried PP powders are presented in Table 1. Among the three drying methods, SOD was found to be best due to low moisture, low carbohydrates and high ash, fats, fiber, and protein contents. Based on this composition, PP peel dried by SOD method was selected for the fortification of FWF for cookies preparation. Results from this study were in line with the work of Mphahlele et al. (32) who dried peel powder by various methods. Romelle et al. (33) reported that the SOD drying method was best in maintaining the proximate composition and other nutritional values of PP powder. The results are also in agreement with the finding of Romelle et al. (33).

**TABLE 1:** Proximate composition, mineral profile, and antioxidant activities of pomegranate peel powder dried by various drying methods.

Drying methods	Proximate composition (%) of pomegranate peel powder					
	**Moisture content**	**Ash**	**Fat**	**Fiber**	**Protein**	**NFE**
SOD	5.55^c^ ± 0.01	4.85^a^ ± 0.01	0.87^a^ ± 0.01	18.48^a^ ± 0.03	4.18^a^ ± 0.07	63.82^c^ ± 0.07
OD	6.56^b^ ± 0.02	4.77^b^ ± 0.01	0.77^b^ ± 0.02	17.15^b^ ± 0.03	3.35^b^ ± 0.05	67.39^b^ ± 0.12
SUD	7.78^a^ ± 0.02	4.6^c^ ± 0.03	0.64^c^ ± 0.03	14.82^c^ ± 0.08	2.96^c^ ± 0.03	71.40^a^ ± 0.06
	**Mineral profile of pomegranate peel powder (mg/100 g)**					
	**Calcium**	**Potassium**	**Iron**	**Zinc**		
SOD	357.1^a^ ± 0.2	161.7^a^ ± 2.48	7.65^a^ ± 0.06	1.83^a^ ± 0.02	–	–
OD	351.5^b^ ± 0.3	154.03^b^ ± 1.45	6.74^b^ ± 0.25	1.64^b^ ± 0.09	–	–
SUD	338.8^c^ ± 0.2	143.66^c^ ± 1.53	5.64^c^ ± 0.23	1.18^c^ ± 0.05	–	–
	**Total phenolic content, flavonoids, and antioxidant activities of pomegranate peel powder**					
	**Phenol (mgGAE g^–1^)**	**Flavonoids (mg quercetin g^–1^)**	**DPPH (%)**			
SOD	30.92^a^ ± 1.12	4.92^a^ ± 1.01	44^b^ ± 0.45		–	–
OD	31.21^a^ ± 1.05	5.06^a^ ± 0.97	42^c^ ± 0.67		–	–
SUD	31.28^a^ ± 0.68	5.08^a^ ± 1.03	48^a^ ± 0.04		–	–

Different letters within each column show significant difference at *p* ≤ 0.05. Each value is a mean of three replicates ± SD. NFE, Nitrogen free extract (carbohydrates); SOD, Solar drying method; OD, Oven drying; SUD, Sun drying method.

### Mineral composition of PP powder

Pomegranate peel powder (PP powder) contains a sufficient amount of minerals. Results showed a significant difference between the three drying methods (Table 1). The SOD powder had the highest mineral contents compared to OD and SUD methods. These results agreed with the findings of Ullah et al. (34) and Ranjitha et al. (35) who reported high mineral contents in PP powder dried by OD method. El-Beltagi (36) evaluate mineral profile of PP powder and stated that PP powder contain high concentration of minerals such as Ca, Mg, Cu, and Fe and recommended to be used as food additive or pharmaceutical industries. Wahab et al. (37) reported that pomegranate peel is renowned by their nutritional minerals which is considered as a good source of micro and macro elements in food and food products. Results of the current study (Table 1) indicates that PP powder is a rich source of minerals and could be the best utilized in food fortification industries to eliminate the diseases like osteoporosis and anemia diseases, from which more than ten million people are suffering (34). Therefore, PP could be a good source of macro and microelements.

### Total phenolic content, total flavonoids, and antioxidant activity of PP powder

Table 1 shows the TPC, TFC, and antioxidant activity (DPPH) of PP powder dried by 3 different methods. No significant difference was found in TPC and TFC among the three drying methods (Table 1). Qabaha et al. (38) reported that pomegranate peel contained 3.5 mg of GAE g^–1^ TPC and 8.3+0.9 mg quercetin g^–1^ TFC. This slight variation in the TPC could be due to the cultivar, climatic or drying temperature difference. Among the three drying methods, high DPPH activity was observed in SUD than OD and SOD drying methods. The phenolic contents act as antioxidants due to their redox properties. The free radical scavenging of phenolic contents is facilitated by their hydroxyl groups. Therefore, the concentration of TPC could be used as rapid screening of anti-oxidative properties by Soobrattee et al. (39). Tolve et al. (40) fortified the wheat flour with grape pomace powders (0, 5, and 10 g/100 g) and prepared fortified bread. These authors stated that fortification wheat flour with grape pomace powder significantly increased the TPC and antioxidant capacity of fortified bread.

Based on the above compositional analysis, PP powder dried by SOD drying method was selected for fortification of WWF and the cookies preparation.

### Proximate composition of FWF fortified with PP powder

#### Moisture content in FWF fortified with PP powder

Table 2 shows the proximate composition of FWF fortified with PP powder dried by a SOD method. PP powder has a great impact on the moisture content of FWF. Moisture contents of FWF was significantly higher while that of PP powder was the lowest. Hence with the increasing concentration of PP powder in FWF, the moisture content significantly (*p* < 0.05) decreased from (11.01 to 9.13) (Table 2). This drastic drop in the moisture content is due to the lower moisture content in PP powder (5.54). The moisture content less than 10% is regarded as safe for the product. The moisture content of flour is critical because a small amount of moisture prevents respiration and microbial activity. Moisture content above than 14 percent promote fungus growth (41). Lipolytic activity occurs when moisture level is high, and results in the loss of nutrients like fat. The decrease in moisture content was attributed to the inclusion of supplements in various proportions as well as the chemical composition.

**TABLE 2:** Proximate analysis (%) of fine wheat flour (FWF) fortified with pomegranate peel (PP) powder.

Treatment	Moisture content	Ash	Crude fat	Crude fiber	Crude protein	NFE
PP powder	5.54^g^ ± 0.01	4.85^a^ ± 0.01	0.8^g^ ± 0.01	18.48^a^ ± 0.01	4.18^g^ ± 0.01	63.82^g^ ± 0.01
FWF-0	11.01^a^ ± 0.01	0.4^g^ ± 0.01	1.06^f^ ± 0.01	1.75^g^ ± 0.01	9.27^a^ ± 0.01	74.43^a^ ± 0.01
FWF-3	10.54^b^ ± 0.01	3.55^f^ ± 0.01	1.35^e^ ± 0.01	6.84^f^ ± 0.01	7.96^b^ ± 0.01	70.47^b^ ± 0.01
FWF-6	10.00^c^ ± 0.01	3.82^e^ ± 0.01	1.52^d^ ± 0.01	7.99^e^ ± 0.01	6.77^c^ ± 0.01	69.86^c^ ± 0.03
FWF-8	9.74^d^ ± 0.01	4.02^d^ ± 0.01	1.68^c^ ± 0.01	10.46^d^ ± 0.01	5.59^d^ ± 0.01	68.52^d^ ± 0.01
FWF-10	9.55^e^ ± 0.01	4.28^c^ ± 0.01	1.84^b^ ± 0.01	12.82^c^ ± 0.01	5.38^e^ ± 0.01	66.10^e^ ± 0.03
FWF-12	9.10^f^ ± 0.01	4.54^b^ ± 0.01	2.04^a^ ± 0.02	15.00^b^ ± 0.01	5.04^f^ ± 0.02	63.94^f^ ± 0.02

Different letters in each column show significant difference at *p* < 0.05. Each value is a mean of three replicates ± SD. PP, Pomegranate peel powder; FWF-0, Fine wheat flour with 0 g PP powder (control); FWF-3, Fine wheat flour with 3 g PP powder; FWF-6, Fine wheat flour with 6 g PP powder; FWF-8, Fine wheat flour with 8 g PP powder; FWF-10, Fine wheat flour with 10 g PP powder; FWF-3 12, Fine wheat flour with 12 g PP powder; NFE, Nitrogen free extract (carbohydrates).

#### Percent ash content in FWF fortified with PP powder

With increasing concentrations of PP powder, the ash content in FWF significantly (*p* < 0.05) increased (Table 2). This could be due to the high ash level in PP powder (4.84%) compared to FWF (0.40%) (Table 2). A similar trend was also observed by Ojha and Sanita (42) when what flour was fortified with mandarin peel powder. During wheat milling, the bran consist the highest ash level is removed, resulting in a decrease in ash content. Therefore, in this study addition of PP powder increased the ash content of the fortified wheat flour.

#### Crude fat in FWF fortified with PP powder

With the addition of PP powder, the fat content of FWF increased significantly (*P* < 0.05). The maximum fat level (2.04 percent) was found when PP powder was added in highest amount (Table 2). Janati et al. (43) reported 4.98% of crude fat in pomegranate peel. Zaker et al. (27) found 1.38% of crude fat in refined wheat flour and 4.41% crude fat when fortified with orange peel powder. Similarly, an increase in fat content was also observed when wheat flour was fortified with various quantities of moringa seed powder (44).

#### Crude fiber in FWF fortified with PP powder

Higher levels of PP powder significantly (*P* < 0.05) increased the fiber content of FWF. The maximum fiber content (18.48%) was found in PP powder, while the lowest fiber content (1.86%) was found in FWF (Table 2). According to Okpala and Akpu (45), increasing the amount of PP powder in FWF greatly enhanced the fiber content.

#### Crude protein in FWF fortified with PP powder

With the addition of PP powder, protein content in FWF decreased significantly (*P* < 0.05) (Table 2). This reduction in protein content could be due to the low protein content in PP powder. A similar trend was also found in the study of Raj and Masih (46) where less protein was observed in citrus peel and high protein was found in wheat flour. Decline in protein content of pomegranate supplemented biscuits were observed by Ismail et al. (20), who stated that pomegranate peel is devoid of protein and fat fraction. Therefore, in current research, reduction in protein content in fortified cookies still fulfill the FAO (47) recommended dietary allowance that is 0.8 g/Kg^–1^d^–1^.

#### Nitrogen free extract (NFE) in FWF fortified with PP powder

High NFE was found in FWF while low NFE was observed in PP powder (Table 2). With increasing levels of PP powder, the NFE content of FWF reduced considerably (*P* ≤ 0.05). Babiker et al. (48) revealed that wheat flour contained a higher concentration of NFE than the mandarin peel powder. Similarly, increasing the concentrations of dried nettle leaves and moringa seed powder reduced NFE content in wheat flour from 367.38–351.43% to 60.53–48.40%, respectively, (43).

### Proximate composition of cookies fortified with PP powder

#### Moisture content in cookies fortified with PP powder

The moisture content of cookies was significantly affected by PP powder. it was found that increasing the concentration of PP Powder results in the reduction of moisture in cookies. In the control sample, maximum moisture content (4.36%) was noted, while lowest moisture content (3.69%) was observed in cookies fortified with 12 g PP powder (Table 3). This shows that fortification has a great effect on the quality of cookies in terms of longer shelf life as Ayo et al. (28) stated that cookies with a moisture content of less than 10% had a longer shelf life and a very low chance of decomposition and spoilage.

**TABLE 3:** Proximate composition (%) of cookies fortified with pomegranate peel (PP) powder.

Treatment	Moisture content	Ash	Crude fat	Fiber	Protein	NFE
CFP-0	4.36^a^ ± 0.02	0.55^f^ ± 0.02	22.12^f^ ± 0.02	1.63^f^ ± 0.01	12.45^a^ ± 0.02	58.87^a^ ± 0.01
CFP-3	4.24^b^ ± 0.02	0.85^e^ ± 0.01	25.03^e^ ± 0.01	1.73^e^ ± 0.02	11.69^b^ ± 0.01	56.75^b^ ± 0.01
CFP-6	4.14^c^ ± 0.03	1.01^d^ ± 0.02	25.63^d^ ± 0.01	2.06^d^ ± 0.02	10.88^c^ ± 0.01	56.28^c^ ± 0.02
CFP-8	4.03^d^ ± 0.01	1.16^c^ ± 0.01	26.09^c^ ± 0.01	2.29^c^ ± 0.02	10.36^d^ ± 0.02	56.04^d^ ± 0.02
CFP-10	3.90^e^ ± 0.02	1.28^b^ ± 0.01	26.72^b^ ± 0.02	2.72^b^ ± 0.02	9.73^e^ ± 0.02	55.64^e^ ± 0.02
CFP-12	3.69^f^ ± 0.02	1.39^a^ ± 0.03	27.06^a^ ± 0.02	2.85^a^ ± 0.02	9.01^f^ ± 0.01	55.44^f^ ± 0.01

Different letters in each column show significant difference at *p* < 005. Each value is a mean of three replicates ± SD. CFP-0, Cookies fortified with 0 g PP powder (control); CFP-3, Cookies fortified with 3 g PP powder; CFP-6, Cookies fortified with 6 g PP powder; CFP-8, Cookies fortified with 8 g PP powder; CFP-10, Cookies fortified with 10 g PP powder; CFP-12, Cookies fortified with 12 g PP powder; NFE, Nitrogen free extract (carbohydrates).

#### Ash content in cookies fortified with PP powder

With increasing concentration of PP powder, ash content in the cookies significantly increased (Table 3). Maximum ash content (1.39) was observed in cookies containing 12 g of PP powder while the lowest ash content was observed in control sample. These results are in line with the work of Mahmoud et al. (49) who found an increase in the ash content from 0.77 to 1.66% in biscuits when fortified with orange peel powder.

#### Crude fat and fiber in cookies fortified with PP powder

Data of crude fat and fiber of cookies is presented in Table 3. Addition of PP powder significantly (*p* ≤ 0.05) increased the crude fat and crude fiber in cookies with few exceptions. Maximum crude fat (27.06%) and crude fiber (2.85%) contents were noted in cookies containing 12 g of PP powder while minimum crude fat and crude fiber were observed in control samples. Ikuomola et al. (50) prepared cookies from wheat flour fortified with malted barley bran increased the fat and fiber content in cookies. In another study, the addition of grape pomace powder (0 to 5 and 0 to 10 g/100 g) to wheat flour increased the total dietary fiber content of fortified bread [Tolve et al. (40)].

#### Protein and NFE contents in cookies fortified with PP powder

Increasing the concentration of PP powder decreased the protein and NFE in cookies (Table 3). The cookies containing 12 g of PP powder resulted in a decrease in the protein from 12.45 to 9.01% and NFE from 58.87 to 55.44% (Table 3). Zaker et al. (27) stated that the wheat flour and orange peel powder cookies in different proportions reduced the amount of protein from 9.88 to 6.8%. Ikuomola et al. (50) found that cookies prepared from wheat flour supplemented with malted barley bran reduced the NFE from 52.70 to 40.05%.

#### Minerals content of cookies fortified with PP powder

With increasing concentration of PP powder, calcium, potassium, zinc, and iron contents in cookies significantly increased (Table 4). This increase in the mineral content in the fortified cookies is due to the high mineral contents in PP powder. This can be seen in Table 4 that in control treatment (without PP powder), the lowest contents of calcium, potassium, iron, and zinc were observed compared to the fortified cookies.

**TABLE 4:** Mineral contents (mg/100 g) of cookies fortified with pomegranate peel (PP) powder.

Treatments	Calcium	Potassium	Iron	Zinc
CFP-0	4.42^f^ ± 0.03	33.91^f^ ± 0.01	0.044^f^ ± 0.001	0.257^f^ ± 0.02
CFP-3	7.84^e^ ± 0.01	38.61^e^ ± 0.01	0.045^e^ ± 0.001	0.262^e^ ± 0.01
CFP-6	10.8^d^ ± 0.01	43.42^d^ ± 0.02	0.046^d^ ± 0.01	0.267^d^ ± 0.01
CFP-8	12.83^c^ ± 0.01	48.41^c^ ± 0.01	0.046^c^ ± 0.001	0.273^c^ ± 0.01
CFP-10	14.71^b^ ± 0.02	54.22^b^ ± 0.02	0.047^b^ ± 0.002	0.278^b^ ± 0.01
CFP-12	17.27^a^ ± 0.01	58.77^a^ ± 0.05	0.048^a^ ± 0.001	0.284^a^ ± 0.01

Different letters in each column show significant difference at *p* < 005. Each value is a mean of three replicates ± SD. CFP-0, Cookies fortified with 0 g PP powder (control); CFP-3, Cookies fortified with 3 g PP powder; CFP-6, Cookies fortified with 6 g PP powder; CFP-8, Cookies fortified with 8 g PP powder; CFP-10, Cookies fortified with 10 g PP powder; CFP-12, Cookies fortified with 12 g PP powder.

Minerals are very important for the human body. Calcium and potassium play a vital role in the body growth and maintenance, a high-potassium diet may aid in the reduction of water retention and blood pressure as well as the prevention of kidney stones and osteoporosis. Zinc plays a significant function in the body’s immune system stimulation and the prevention of oxidative stress. Iron is important for the body to prevent anemia. Therefore, fortification of cookies is one of the best approach to fulfill the body requirements and prevent from these diseases. Generally, fruit peel is rich in minerals contents compared to pulp (51). Other researchers also worked on fortifications. For example, Khan et al. (22) enhanced the nutritional values and minerals profile of leavened and unleavened bread prepared from the whole wheat flour fortified with eggshells and chicken bones powders. Pomegranate peels are considered as a good source of minerals (Zn, Fe, Mg, K, and Ca) and can provide sufficient micronutrients than non-supplemented cookies (52). The most satisfactory and sustainable supplement carriers are cookies in bakery products. Wheat flour is lacking enough minerals, fibers, and antioxidants (53). To overcome the nutritional demands of consumer and enhance the mineral contents of bakery products, supplementation of pomegranate peel powders to cookies flour is easy way as PP powder are rich in these natural compounds (54). Tessera et al. (55) and Bolarinwa et al. (44) prepared cookies supplemented with of different concentration of moringa leave and seed powder and reported that by increasing the concentration moringa leaves and seed powder in wheat flour, mineral contents (Mg, K, Fe, and Ca) in the supplemented cookies significantly increased.

#### Physical properties (weight, width, thickness, and spread ratio) of cookies fortified with PP powder

Table 5 shows the weight, width, thickness and spread ratio values of the prepared cookies fortified with PP powder. Weight, width and spread ratio of cookies significantly *p* < 005 increased with increasing the concentration of PP powder while the thickness of the cookies decreased. Highest weight, width and spread ratio and lowest thickness values were observed in cookies containing 12 g of PP powder, while on the other hand, the lowest weight, width, spread ratio and highest thickness values were observed in control treatment (Table 5). Increase in weight, width, and spread ratio could be due to the water absorption, load increasing and dough making process (56). Ho and Latif (29) stated that an increase in the width and decrease in the thickness are due to the protein dilution during the heating process of dough. Protein in flour generates a web-like structure in cookies, which is responsible for irreversible dough expansion. As a result, adding higher quantities of PP powder resulted in a larger diameter cookie with less thickness and vice versa. Increase in the spread ratio was also reported by Ho and Latif (29) when cookies were prepared from wheat flour fortified with pitaya peel powder.

**TABLE 5:** Physical properties of cookies fortified with pomegranate peel (PP) powder.

Treatments	Weight (g)	Width (mm)	Thickness (mm)	Spread ratio
CFP-0	15.22^f^ ± 0.01	44.83^f^ ± 0.37	8.34^a^ ± 0.01	5.37^a^ ± 0.05
CFP-3	16.46^e^ ± 0.01	46.96^e^ ± 0.63	8.22^b^ ± 0.01	5.72^b^ ± 0.03
CFP-6	17.35^d^ ± 0.02	48.50^d^ ± 0.45	8.07^c^ ± 0.01	6.00^c^ ± 0.06
CFP-8	18.31^c^ ± 0.01	49.57^c^ ± 0.32	7.94^d^ ± 0.01	6.24^d^ ± 0.03
CFP-10	19.81^b^ ± 0.01	50.73^b^ ± 0.30	7.81^e^ ± 0.01	6.49^e^ ± 0.05
CFP-12	20.61^a^ ± 0.01	51.92^a^ ± 0.35	7.65^f^ ± 0.01	6.79^f^ ± 0.05

Different letters in each column show significant difference at *p* < 005. Each value is a mean of three replicates ± SD. CFP-0, Cookies fortified with 0 g PP powder (control); CFP-3, Cookies fortified with 3 g PP powder; CFP-6, Cookies fortified with 6 g PP powder; CFP-8, Cookies fortified with 8 g PP powder; CFP-10, Cookies fortified with 10 g PP powder; CFP-12, Cookies fortified with 12 g PP powder.

#### Sensory attributes of cookies fortified with PP powder

**FIGURE 2:**
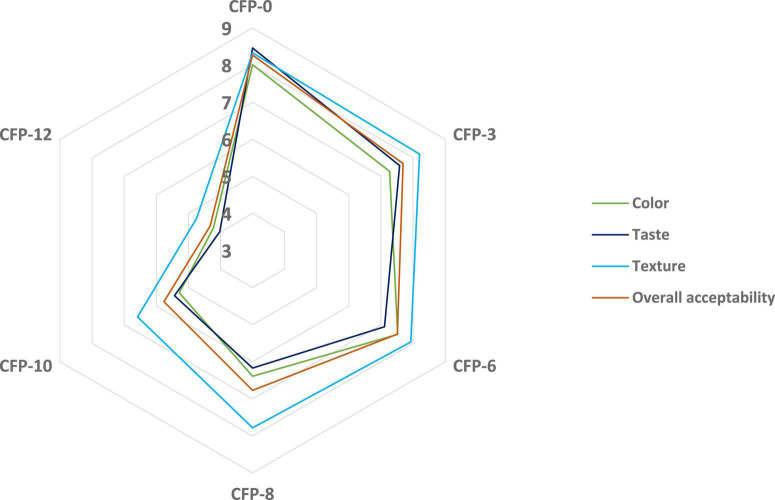
Sensory attributes of fortified cookies using 9-points hedonic scale (1 = extremely dislike, 2 = very much dislike, 3 = moderately dislike, 4 = dislike-slightly, 5 = neither-like nor-dislike, 6 = like-slightly, 7 = like-moderately, 8 = like-very much, 9 = like-extremely). CFP-0 = Cookies fortified with 0 g PP powder (control), CFP-3 = Cookies fortified with 3 g PP powder, CFP-6 = Cookies fortified with 6 g PP powder, CFP-8 = Cookies fortified with 8 g PP powder, CFP-10 = Cookies fortified with 10 g PP powder, CFP-12 = Cookies fortified with 12 g PP powder.

**FIGURE 3:**
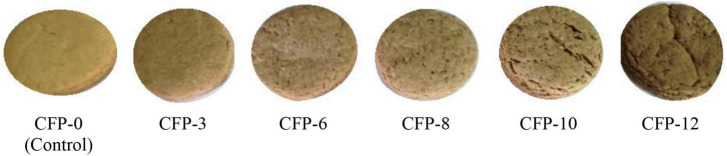
Pictures of cookies. CFP-0 = Cookies fortified with 0 g PP powder (control), CFP-3 = Cookies fortified with 3 g PP powder, CFP-6 = Cookies fortified with 6 g PP powder, CFP-8 = Cookies fortified with 8 g PP powder, CFP-10 = Cookies fortified with 10 g PP powder, CFP-12 = Cookies fortified with 12 g PP powder.

Sensory analysis such as color, texture, taste and overall acceptability values are presented in Table 6. PP powder significantly (*P* < 0.05) affected the sensory attributes. With the addition of PP powder, the color, texture, taste and overall acceptability values of cookies decreased (Figures 2, 3). Cookies without PP powder (control) obtained the highest score of color, texture, taste, and overall acceptability. A decrease in these sensory attributes could be due to the soluble tannins in PP powder which provided a bitter taste (29). Ikuomola et al. (50) reported that cookies prepared from wheat flour supplemented with malted barley bran (5, 10, 20, and 50%) and found that increase in substitution of barley bran than 5% significantly affected the consumer acceptability for color, texture, taste, and overall acceptability, whereas the bitter taste and dark brown color decrease the consumer acceptance of cookies. Bala et al. (19) prepared cookies from cassava flour supplemented with water chestnut flour (0–100%) and reported a significant decrease in the sensory parameter such as color, taste, flavor and overall acceptability. Similar results were also reported by Mir et al. (57) when in cookies supplemented with water chestnut flour. Although the cookies were acceptable to the panelist, however, further work is recommended on the PP powder to improve its taste before used for fortification purposes.

**TABLE 6:** Sensory attributes of cookies fortified with pomegranate peel (PP) powder.

Treatments	Color	Taste	Texture	Overall acceptability
CFP-0	8.01^a^ ± 0.03	8.46^a^ ± 0.01	8.33^a^ ± 0.02	8.27^a^ ± 0.01
CFP-3	7.27^b^ ± 0.05	7.58^b^ ± 0.07	8.20^b^ ± 0.01	7.69^b^ ± 0.03
CFP-6	7.52^c^ ± 0.02	7.11^c^ ± 0.09	7.93^c^ ± 0.02	7.51^c^ ± 0.01
CFP-8	6.39^d^ ± 0.06	6.17^d^ ± 0.14	7.78^d^ ± 0.02	6.77^d^ ± 0.03
CFP-10	5.28^e^ ± 0.17	5.43^e^ ± 0.31	6.58^e^ ± 0.03	5.76^e^ ± 0.09
CFP-12	4.22^f^ ± 0.22	4.02^f^ ± 0.03	4.74^f^ ± 0.06	4.32^f^ ± 0.08

Different letters in each column show significant difference at *p* < 005. Each value is a mean of three replicates ± SD. CFP-0, Cookies fortified with 0 g PP powder (control); CFP-3, Cookies fortified with 3 g PP powder; CFP-6, Cookies fortified with 6 g PP powder; CFP-8, Cookies fortified with 8 g PP powder; CFP-10, Cookies fortified with 10 g PP powder; CFP-12, Cookies fortified with 12 g PP powder.

## Conclusion

Pomegranate peel (PP) powder is a rich source of macro, micronutrient and bioactive compounds and minerals. In this study, PP powder was dried by different methods such as solar, oven and sun drying methods. Then proximate compositions, minerals profile, TPC, TFC, and DPPH activity of the PP powder were studied to select the best dying powder with high nutritional values for fortification purpose. Solar dried PP powder was then incorporated in FWF and proximate composition was determined. Based on the results obtained, SOD dried PP powder were incorporated in different concentrations into FWF with the addition of other ingredients and cookies were prepared. PP powder significantly affected the compositional analysis, enhanced minerals contents of the cookies. PP powder also positively affected the physical qualities of cookies such as weight, width, thickness and spread ratio. Sensory analysis like color, texture, taste and overall acceptability of the cookies although decreased, however, the cookies were in the acceptable to the consumer’s panel. PP powder at 6 and 8 g concentration was found the best among the other concentrations in terms of sensory evaluations. Therefore, these concentrations are recommended for commercial application in baking industries to full fill the dietary requirement of the people.

## Data availability statement

All data obtained and analyzed in this study are included in the manuscript.

## Author contributions

AM, JP, and KD: conceptualization. AM and MS: methodology and investigation. MK, RA, and GN: software. KD and JP: validation and supervision. AM: formal analysis and writing—original draft preparation. KD, JP, and MK: resources. HU: data curation. MK, AM, and HU: writing—review and editing. RA: visualization. KD: project administration. JP: funding acquisition. All authors read and agreed to the published version of the manuscript.
